# The role of VEGF-C/D and Flt-4 in the lymphatic metastasis of early-stage invasive cervical carcinoma

**DOI:** 10.1186/1756-9966-28-98

**Published:** 2009-07-09

**Authors:** Hao Yu, Shiqian Zhang, Renhua Zhang, Linlin Zhang

**Affiliations:** 1 Department of Obstetrics and Gynecology, Qilu Hospital of Shandong University, Ji'nan 250012, PR China; 2 Department of Obstetrics and Gynecology, Fourth People's Hospital of Jinan, Ji'nan 250031, PR China

## Abstract

**Background:**

To investigate the role of vascular endothelial growth factors (VEGF)-C/D and their receptor Flt-4 in the lymphatic metastasis of early-stage invasive cervical carcinoma.

**Methods:**

Immunohistochemical (IHC) staining with the antibodies against VEGF-C, VEGF-D, and Flt-4 was used to examine the expression of them in 97 cases of early-stage cervical carcinoma (Ia-IIa). Meanwhile, the lymphatic vessel density (LVD) was measured using the antibody against lymphatic vessel endothelial hyaluronan receptor-1 (LYVE-1). We then analyzed the correlation between Flt-4-positive vessel density (FVD), LVD and clinicopathological features of the tumors.

**Results:**

(1) The positive rates of VEGF-C, VEGF-D, and Flt-4 were 57.7%, 60.8%, and 52.6% in the cervical tumor samples, respectively. (2) The expression levels of VEGF-C, VEGF-D, and Flt-4 were significantly correlated with lymphatic metastasis and lymphatic vessel invasion. LVD was significantly associated with lymph node metastasis and lymphatic vessel invasion. On the other hand, FVD was strongly associated with clinical staging. (3) The expression levels of VEGF-C and VEGF-D were significantly correlated with LVD and FVD, while Flt-4 levels showed no correlation with LVD or FVD.

**Conclusion:**

VEGF-C/D and Flt-4 may play an important role in the process of lymphatic metastasis of early-stage invasive cervical carcinoma through paracrine and autocrine mechanisms.

## Background

Cervical cancer is the most common malignant gynecological cancer, and lymphatic metastasis is one of the most important metastatic routes of this cancer. The involvement of the lymphatic node is usually one of the factors predicting the prognosis. Along with the development of specific markers of lymphatic endothelium [1] and the improvement of isolation techniques for lymphatic endothelial cells [2], the role of tumor lymphangiogenesis in the metastasis of early-stage cervical carcinoma is gradually becoming a research focus. Even so, the mechanism of the tumor lymphatic metastasis is still largely unknown and there is a great deal of debate over various aspects of research in the field. In this study, we employed immunohistochemical (IHC) staining to detect the expression of VEGF-C, VEGF-D, and Flt-4 in early-stage cervical carcinoma (Ia-IIa). The lymphatic vessel endothelial hyaluronan receptor-1 (LYVE-1), the labeled lymphatic vessel density (LVD) and Flt-4-positive vessel density (FVD) were also measured and analyzed relative to the clinicopathological features of the tumors. Our study explored the roles of VEGF-C, VEGF-D, and FLt-4 in the lymphatic metastasis of early-stage cervical cancer.

## Materials and methods

### Patients and tissue samples

Patients with cervical carcinoma who were treated between September 2007 and February 2009 were enrolled in this study (*n *= 97). The tissue samples were obtained at the time of surgery from the Department of Gynecology, Qilu Hospital, Shandong University. Samples and clinical data were collected after informed consent was obtained. Tissues were fixed with 4% paraformaldehyde and paraffin-embedded for further analysis. The pathological examination verified that no radio- or chemotherapy was received before surgery. Our study was approved by the Ethics Committee of Shandong University. All patients with early-stage invasive cervical cancer were staged according to the 2000 International Federation of Gynecology and Obstetrics (FIGO) staging system. Sixteen of the patients had cervical cancer classified as FIGO stage Ia, 33 as FIGO stage Ib, and 48 as FIGO stage IIa. Based on the analysis of cellular differentiation, 21 cases were HG_1_, 31 were HG_2_, and 45 were HG_3_. Of all the cases, 81 were squamous cell carcinomas and 16 were adeno-carcinomas. All the patients received pelvic or para-abdominal aortic lymphadenectomy and in total 2376 lymph nodes were dissected (mean 24.5, median 24.0). A histological review confirmed that 30 cases (30.9%) showed lymph node metastasis and 75 lymph nodes were metastasis positive (mean 2.5, median 2). The age of the patients varied from 26 to 70, with a median value of 42. Of all the patients, 68 were premenopausal and 29 were postmenopausal. The standard for lymphatic vessel invasion was the detection of cancer cells in the cavity of the lymphatic vessel by light microscopy. By this standard, 39 cases showed lymphatic vessel invasion and 58 were negative. All tissue specimens and slides were examined by experienced pathologists.

### Reagents

The reagents used in this study included: rabbit anti-human VEGF-C polyclonal antibody from Zhongshan Goldenbridge Biotech (Beijing, China; catalog no. ZA-0266, 1:50 dilution); rabbit anti-human VEGF-D polyclonal antibody from Boster Inc. (Wuhan, Hubei, China; catalog no. BA1461, 1:100 dilution); rabbit anti-human Flt-4 polyclonal antibody from Abcam (Cambridge, MA, USA; catalog no. ab27278, 1:200 dilution); rabbit anti-human LYVE-1 polyclonal antibody from Abcam (Cambridge, MA, USA; catalog no. ab36993, 1:80 dilution); and an immunohistochemistry SP kit from Jingmei Inc. (Shanghai, China; catalog no. LHK612).

### Immunohistochemistry

Immunohistochemistry (IHC) was conducted using the immunohistochemistry SP kit from Jingmei Inc. according to the manufacturer's instructions. Briefly, serial section slides of 5 μm were obtained from the paraffin-embedded specimens. After regular de-paraffin and re-hydration, the slides were placed in an antigen retrieval solution (pH 6.0) and heated in a microwave oven for 10 min at 95°C. Next, the slides were incubated in a 3% hydrogen peroxide-methanol solution for 10 min to remove endogenous peroxidase. IHC staining was performed as follows: nonspecific binding was blocked with 10% goat serum; the slide was incubated for 1 h with primary antibodies, followed by incubation for 30 min with a biotin-labeled secondary antibody; and subsequently the slide was incubated for 30 min with horseradish peroxidase-labeled streptavidin. Color was developed using DAB, and the slide was counterstained with hematoxylin. Finally, the slides were mounted and coverslipped with resinene. Negative control slides were stained with PBS instead of the primary antibodies. Breast cancer slides were used as a positive control.

VEGF-C, VEGF-D, and Flt-4 positive cells showed brown-yellow particles in their cytoplasm. According to the method described by Jüttner et al.[3], the samples were classified as follows: - (no positive cell), + (0–5% positive cell), ++ (5–50% positive cell), +++ (>50% positive cell). Among these, ++ and +++ samples were determined to have a positive expression. LVD and FVD were determined according to the methods previously described by Weidner et al. [4]. Briefly, the slides were scanned on a low-power microscope and areas with the highest positively stained vessel density, called hot spots, were identified. The number of positively stained lymphatic vessels in five high-power fields in the selected areas was counted. LVD and FVD were determined as the mean value of vessel counts.

### Statistical Analysis

All statistical calculations were performed using SPSS (version 13.0, Chicago, IL USA). LVDs and FVDs were expressed as means ± SD. The statistical methods used included the t-test, the one-way ANOVA test, and the Chi-square test. Differences were considered to be statistically significant when P < 0.05.

## Results

### Expression of VEGF-C, VEGF-D and Flt-4 in cervical cancer tissue

**Figure 1:**
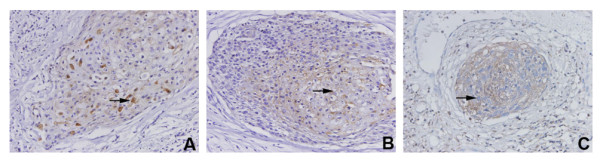
**The expression of VEGF-C (A), VEGF-D (B), and Flt-4 (C) in cervical carcinoma tissues**. A. IHC detection of VEGF-C (→) ×400; B. IHC detection of VEGF-D (→) ×400; and C. IHC detection of Flt-4 (→) ×400.

The IHC signals of VEGF-C, VEGF-D, and their receptor Flt-4 were mostly localized in the cytoplasm of the cancer cell in the examined cervical carcinoma samples and the positively stained cells showed a brown-yellow color in the cytoplasm. The positive rates were 57.7% (56 out of 97) for VEGF-C, 60.8% (59 out of 97) for VEGF-D, and 52.6% (51 out of 97) for Flt-4 (Figure 1A).

Next, we analyzed the correlation between the VEGF-C, VEGF-D, and Flt-4 signals and the pathological features. The results from statistical analyses showed that the expression of both VEGF-C and VEGF-D were positively correlated with lymph node metastasis and lymphatic vessel invasion, but expression was not associated with menopause, tumor size, stromal invasion, FIGO stage, histological grade, or histological types. Similarly, Flt-4 expression was only associated with lymph node metastasis and lymphatic vessel invasion, but not with the other factors analyzed (Table 1).

**Table 1:** Correlation of expression of VEGF-C, VEGF-D, and Flt-4 in cervical cancer tissues with clinicopathological parameters

Variables	*n*	VEGF-C			VEGF-D			Flt-4		
		(+)	(-)	*P*	(+)	(-)	*P*	(+)	(-)	*P*
Catamenia										
Premenopause	68	37	31	NS	42	26	NS	33	35	NS
Postmenopause	29	19	10		17	12		18	11	
Tumor size (cm)										
≤4	61	36	25	NS	35	26	NS	30	31	NS
>4	36	20	16		24	12		21	15	
Stromal invasion										
≤2/3	40	22	18	NS	27	13	NS	24	16	NS
>2/3	57	34	23		32	25		27	30	
FIGO stage										
I a	16	10	6	NS	7	9	NS	9	7	
I b	33	18	15		22	11		18	15	
II a	48	28	20		30	18		24	24	
Histological grade										NS
HG_1_	21	9	12	NS	12	9	NS	10	11	
HG_2_	31	18	13		20	11		15	16	
HG_3_	45	29	16		27	18		26	19	
Lymph node metastasis										
Negative	67	33	34	0.012	35	32	0.010	30	37	0.022
Positive	30	23	7		24	6		21	9	
LVI										
Negative	39	16	23	0.006	18	21	0.015	14	25	0.007
Positive	58	40	18		41	17		37	21	
Histological cell type										
SCC	81	46	35	NS	50	31	NS	43	38	NS
ADE	16	10	6		9	7		8	8	

Abbreviations: HG, histological grade; LVI, lymphatic vessel invasion; SCC, squamous cell carcinoma; and ADE, adenocarcinoma. P, chi-square test.

### Lymphatic vessel density and Flt-4 positive vessel density

**Figure 2:**
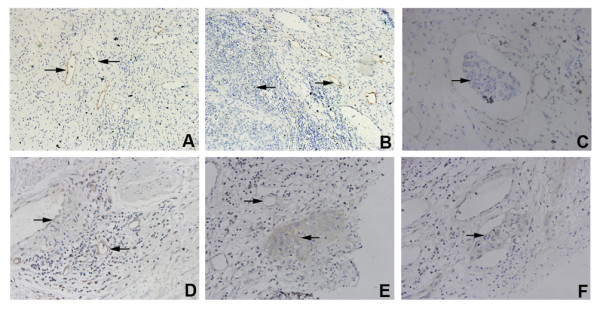
**Morphological features of LYVE-1 positive lymphatic vessels and Flt-4 positive vessels in cervical cancer tissues**. A. The LYVE-1 positive lymphatic vessels (→) were clearly different from blood vessels (←) ×200; B. The LYVE-1 positive lymphatic vessels (→) were mainly distributed in the paratumor stromal tissue (←) ×400; C. Some LYVE-1 positive lymphatic vessels contained invaded tumor cells (→) ×400; D. Some Flt-4 positive vessels were similar to blood vessels in their morphology (→), and others were similar to lymphatic vessels (←) ×400; E. The Flt-4 positive vessels (→) were mainly distributed in the paratumor stromal tissue (←) ×400; and F. Some Flt-4 positive vessels contained invaded tumor cells (→) ×400.

Analysis under a light microscope showed that the LYVE-1 positive vessels were composed of a single layer of cells with a large nucleus extruding towards the lumen face. The basal and lumen faces were both stained in a brown-yellow color, which was clearly different from blood vessels (Figure 2A). These lymphatic vessels were mostly distributed in the stromal tissue surrounding the tumor (Figure 2B), and tumor cells were observed in some LYVE-1 positive lymphatic vessels (Figure 2C). Under the light microscope, some of the Flt-4 positive vessels showed blood vessel morphology and the others showed lymphatic vessel morphology (Figure 2D). Most of the Flt-4 positive vessels were distributed in the stromal tissue surrounding the tumors (Figure 2E). Some of the Flt-4 positive lymphatic vessels contained tumor cells which were also Flt-4 positive (Figure 2F).

We also analyzed the LVD and FVD. LVD was positively correlated with lymph node metastasis and lymphatic vessel invasion of the tumor, but not with menopause, tumor size, depth of stromal invasion, FIGO stage, histological grade, or histological type. FVD was positively associated with FIGO stage, but not with the other pathological features (Table 2).

**Table 2:** Association of LVD and FVD with clinical and pathological parameters

Variables	*n*	LVD		FVD	
			
		mean ± SD	*P*	mean ± SD	*P*
Catamenia					
Premenopause	68	17.00 ± 1.63	NS	25.97 ± 1.48	NS
Postmenopause	29	16.33 ± 1.44		25.41 ± 1.83	
Tumor size (cm)					
≤4	61	16.66 ± 1.26	NS	26.32 ± 1.92	NS
>4	36	17.06 ± 1.22		26.97 ± 1.84	
Stromal invasion					
≤2/3	40	16.29 ± 0.86	NS	25.82 ± 1.66	NS
>2/3	57	16.69 ± 1.23		26.02 ± 1.70	
FIGO stage					
a	16	16.43 ± 1.40	NS*	25.09 ± 1.49	0.032*
b	33	17.07 ± 1.49		25.21 ± 1.62	
a	48	17.10 ± 1.52		26.10 ± 1.85	
Histological grade					
HG_1_	21	16.86 ± 1.57	NS*	25.43 ± 1.98	NS*
HG_2_	31	17.15 ± 1.14		26.08 ± 1.75	
HG_3_	45	17.24 ± 1.37		25.76 ± 1.37	
Lymph node metastasis					
Negative	67	17.15 ± 1.49	0.025	25.70 ± 1.84	NS
Positive	30	17.93 ± 1.70		26.33 ± 1.82	
LVI					
Negative	39	16.49 ± 1.46	0.001	25.97 ± 1.66	NS
Positive	58	17.66 ± 1.82		26.50 ± 1.74	
Histological cell type					
SCC	81	16.76 ± 1.62	NS	25.78 ± 1.64	NS
ADE	16	17.25 ± 1.26		26.00 ± 1.15	

Abbreviations: HG, histological grade; LVI, lymphatic vessel invasion; SCC, squamous cell carcinoma; ADE, adenocarcinoma, LVD, lymphatic vessels density; and FVD, Flt-4-positive vessel density. P, t-test; P*, one-way ANOVA test.

We also cross-analyzed the correlation of expression levels of VEGF-C, VEGF-D, and Flt-4 with LVD and FVD. We found that the expression of VEGF-C and VEGF-D was correlated with LVD and FVD, but the expression of Flt-4 was not associated with LVD and FVD (Table 3).

**Table 3:** Association of expression of VEGF-C, VEGF-D, and Flt-4 with LVD and FVD in cervical carcinoma

		*n*	LVD	*P*	FVD	*P*
VEGF-C	(+)	56	18.10 ± 0.85	0.026	27.05 ± 0.86	0.020
	(-)	41	17.87 ± 1.02		26.60 ± 1.00	
VEGF-D	(+)	59	17.88 ± 0.94	0.046	26.82 ± 1.28	0.022
	(-)	38	17.49 ± 0.91		26.18 ± 1.38	
Flt-4	(+)	51	17.15 ± 1.01	NS	25.63 ± 1.66	NS
	(-)	46	16.77 ± 1.32		26.06 ± 1.47	

Abbreviations: LVD, lymphatic vessels density; and FVD, Flt-4-positive vessel density. P, chi-square test.

## Discussion

Within research exploring the mechanisms of tumor metastasis, there are more and more studies examining the relationship between tumor lymphangiogenesis and tumor metastasis. The important role of lymphatic vessels in tumor metastasis, and especially solid tumor metastasis, has been recognized gradually. Clinicopathological research has demonstrated that the earliest path for solid tumor metastasis is regional spreading to the lymph nodes through lymphatic vessels. The regulatory mechanism for lymphangiogenesis in tumors is complicated and there are multiple factors involved. Among them, VEGF-C, VEGF-D, and Flt-4 were thought to be the major regulatory factors for tumor lymphangiogenesis, and likely play an important role in the lymphatic tumor metastasis. Animal tumor models with overexpression of VEGF-C or VEGF-D have shown that the lymphatic endothelial cells proliferated quickly in tumor tissues, LVD increased significantly, and lymph node metastasis was also enhanced. Jeltsch et al. [5] reported that high-expression of VEGF-C in a K14-VEGF-C transgenic mice model promoted lymphatic endothelial cell proliferation and enlargement of lymph vessel cavity in the dermis of mice. Von Marschall et al. [6] transplanted human pancreatic duct cancer cells that overexpressed VEGF-D into nude mice and detected LYVE-1 positive lymphatic vessels. They found that VEGF-D significantly induced the lymphangiogenesis, increased LVD in the tumor tissues, and was closely related to the significantly increased lymphatic vessel invasion and lymph node metastasis. In our study, we showed that VEGF-C, VEGF-D, and Flt-4 were significantly correlated with lymph node metastasis and lymphatic vessel invasion. Meanwhile, we used a polyclonal LYVE-1 antibody to label lymphatic vessels. Since the measurement of lymphatic vessel density can be quite subjective, a strategy was applied in which the lymphatic vessel density was measured by two expert pathologists, who were blinded for clinical data. LVD counting demonstrated that LVD was associated with lymph node metastasis and lymphatic vessel invasion and was closely related to levels of VEGF-C and VEGF-D, which is consistent with previous clinical studies [7-9]. The underlying mechanism may be secretion of VEGF-C and VEGF-D by tumor cells, which then function through the receptor tyrosine kinase Flt-4 in lymphatic endothelial cells in a paracrine manner and promote endothelial proliferation, differentiation and cavity formation. These newly generated lymphatic vessels in tumor tissues are structurally similar to the physiological lymphatic vessels, but occur in large numbers and in thin walls. These features provide more paths for tumor cell infiltration and facilitate tumor metastasis.

The mechanism of tumor lymphangiogenesis is complicated and involves an interaction between tumor cells and lymphatic endothelial cells. It has been shown that increased lymphatic vessels in tumor not only provide paths for tumor cell metastasis, but also release proteases under the stimulation of VEGF-C and promote basal membrane infiltration of tumor cells [10]. On the other hand, VEGF-C also changed the adherent features and expression of surface chemo-attractants and receptors, affected the process by which tumor cells enter lymphatic vessels and therefore actively promote the tumor lymphatic metastasis [11].

Although increased LVD provides more metastatic pathways and plays an important role in tumor lymphatic metastasis, the process of tumor lymphatic metastasis is complicated and has multiple steps, including tumor cell migration, degradation of extracellular matrix, and relocation. Migration and invasion of tumor cells are prerequisites for tumor metastasis and infiltration. As the receptor for VEGF-C and VEGF-D, Flt-4 is expressed in not only the lymphatic endothelial cells, but also in the liver and spleen blood sinus, during injury repair, and in newly generated tumor blood vessel endothelium. Recent studies have shown that Flt-4 was also expressed in many types of tumor cells [12,13] and played an important role in tumor lymphatic metastasis and tumor progression by promoting tumor cell proliferation, growth, and migration [14].

Su et al. [15] used in vitro migration and invasion methods and found that some tumor cells with a strong invasion ability, such as cervical carcinoma cell SiHa, had not only a high expression level of VEGF-C, but also a high level of Flt-4. Human recombinant VEGF-C (Cys 156 Ser) protein could promote the migration and invasion of tumor cells. Application of recombinant Flt-4/Fc blocked signaling of VEGF-C and also significantly decreased tumor migration and invasion. This suggested that Flt-4/Fc enhances lymphangiogenesis by affecting paracrine signaling, and that VEGF-C, VEGF-D and Flt-4 might also have an autocrine function in promoting tumor cell migration and invasion, which could eventually lead to tumor lymphatic metastasis. Van et al. [16] found that in the transition from localized cervical epithelial neoplasia to metastatic cervical carcinoma, the expression of VEGF-C, VEGF-D, and Flt-4 increased gradually. Therefore, it was speculated that VEGF-C, VEGF-D and Flt-4 could be involved in the process of phenotypic transition to lymphangiogenesis and could facilitate lymphatic metastasis in the early stages of cervical cancer. In addition, Masood et al. [17] found that VEGF-C and VEGFR-3 activation promoted the growth of malignant pleural endotheliomas. Consistently, the application of antisense oligos against VEGF-C, recombinant VEGFR-3/Fc, or VEGFR-3 antibody to inhibit VEGF-C/VEGFR-3 signaling led to a significantly lower survival of malignant pleural endotheliomas cells. In the current study, we found that in cervical carcinoma, Flt-4 was expressed not only in blood vessel and lymphatic vessel endothelial cells, but also in tumor cells, and that the level of Flt-4 was positively correlated with lymph node metastasis and lymphatic vessel infiltration. This is inconsistent with the results from a previous study by Jüttner et al. [3]. One possible reason for this inconsistency is that there are multiple receptors of VEGF-C and VEGF-D and their expression is heterogeneous in different tumor cells. Except for Flt-4, VEGFR-2, NRP-1 and NRP-2 can all function as receptors for VEGF-C and VEGF-D [18]. Therefore, the roles of VEGF-C, VEGF-D, and Flt-4 in the progress of tumors are omnifarious and the underlying mechanisms of these growth factors need to be further studied.

Our research showed that the specificity of Flt-4 as a lymphatic vessel marker was not high. Some of the Flt-4 positive vessels were morphological blood vessels and other vessels were lymphatic vessels. We found that FVD was positively associated with the FIGO stage of cervical cancer, but was not related to the other clinicopathological features including histological grade, lymph node metastasis, or lymphatic vessel infiltration. In addition, we found that FVD was correlated with the expression of VEGF-C and VEGF-D. This is inconsistent with Yasuoka et al. [19]. The VEGF receptor tyrosine kinase family includes VEGFR-1, VEGFR-2, and VEGFR-3. VEGF-1 and VEGF-2 are primarily expressed in blood vessel endothelial cells and are involved in tumor angiogenesis. Since Flt-4 is expressed in the endothelial cells of blood vessels and lymphatic vessels, VEGF-C, VEGF-D, and Flt-4 may also play important roles in tumor angiogenesis [20].

In summary, our results indicated that VEGF-C, VEGF-D, and Flt-4 may promote tumor lymphangiogenesis and may provide a spreading route for tumor metastasis through a paracrine mechanism. On the other hand, they may function in an autocrine manner to enhance tumor cell migration and invasion and may therefore play an important role in the lymphatic vessel metastasis of early-stage cervical carcinoma.

## Conflicting interests

The authors declare that they have no competing interests.

## Authors' contributions

Hao Yu carried out study design, literature research, experimental studies, data acquisition, data analysis, statistical analysis and manuscript preparation. Shiqian Zhang was the guarantor of integrity of the entire study. Renhua Zhang and Linlin Zhang participated in literature research, data analysis and manuscript editing. All authors read and approved the final manuscript.
